# Comparison of Numerical Simulations to Experiments for Atomization in a Jet Nebulizer

**DOI:** 10.1371/journal.pone.0078659

**Published:** 2013-11-11

**Authors:** Nicolas Lelong, Laurent Vecellio, Yann Sommer de Gélicourt, Christian Tanguy, Patrice Diot, Alexandra Junqua-Moullet

**Affiliations:** 1 DTF Aerodrug, Faculté de Médecine, Université François Rabelais, Tours, France; 2 Centre d’Etudes des Pathologies Respiratoires INSERM U1100/EA 6305, Faculté de Médecine, Université François Rabelais, Tours, France; 3 ANSYS France, Montigny le Bretonneux, France; 4 Dantec Dynamics, Nozay, France; 5 CEA Le Ripault, Monts, France; University Paul Sabatier, France

## Abstract

The development of jet nebulizers for medical purposes is an important challenge of aerosol therapy. The performance of a nebulizer is characterized by its output rate of droplets with a diameter under 5 µm. However the optimization of this parameter through experiments has reached a plateau. The purpose of this study is to design a numerical model simulating the nebulization process and to compare it with experimental data. Such a model could provide a better understanding of the atomization process and the parameters influencing the nebulizer output. A model based on the Updraft nebulizer (Hudson) was designed with ANSYS Workbench. Boundary conditions were set with experimental data then transient 3D calculations were run on a 4 µm mesh with ANSYS Fluent. Two air flow rate (2 L/min and 8 L/min, limits of the operating range) were considered to account for different turbulence regimes. Numerical and experimental results were compared according to phenomenology and droplet size. The behavior of the liquid was compared to images acquired through shadowgraphy with a CCD Camera. Three experimental methods, laser diffractometry, phase Doppler anemometry (PDA) and shadowgraphy were used to characterize the droplet size distributions. Camera images showed similar patterns as numerical results. Droplet sizes obtained numerically are overestimated in relation to PDA and diffractometry, which only consider spherical droplets. However, at both flow rates, size distributions extracted from numerical image processing were similar to distributions obtained from shadowgraphy image processing. The simulation then provides a good understanding and prediction of the phenomena involved in the fragmentation of droplets over 10 µm. The laws of dynamics apply to droplets down to 1 µm, so we can assume the continuity of the distribution and extrapolate the results for droplets between 1 and 10 µm. So, this model could help predicting nebulizer output with defined geometrical and physical parameters.

## Introduction

Aerosol therapy consists in delivering drugs through respiratory airways. The drug is produced as a liquid or solid aerosol of micrometric particles, making it able to reach different areas in the respiratory tract. The main advantage of the technique is to target directly the affected area, depending on the particle size, thus increasing drug efficacy and reducing the risk of side-effects. Many devices exist for that purpose, functioning upon various techniques. This study is focused on one of these devices, the jet nebulizer, which uses pressurized air to break up the liquid drug into small droplets. Unlike other usual aerosol devices such as pressurized Metered Dose Inhalers or Dry Powder Inhalers, which are preloaded with the drug, the nebulizer has to be filled with a liquid formulation of the drug to be administered. It is nowadays widely used because of its reasonably low cost and its assumed potential for higher performances: high liquid flow rate and small particle size. Actually, a relatively low fraction (10%) of the delivered dose can actually reach the deep lung [1]. The deposited fraction of the aerosol is made of a high quantity of droplets with an aerodynamic diameter, smaller than 5 µm [2]. The aerodynamic diameter is defined as the diameter of the spherical particle with a density equal to one and the same velocity as the droplet. As the droplet size distribution produced by a nebulizer is polydisperse, only a fraction of the produced aerosol fits that criterion. Furthermore, the limited output implies a long nebulization time, up to 20 minutes. The physical processes occurring within this device are still not completely understood. Influence of various parameters of the liquid (viscosity and surface tension) and the gas (pressure and velocity) on the particle size have already been demonstrated [3], [4] but the development stays empirical, restricting the optimization potential of the device. No major breakthrough in terms of performance has been made recently, and new ways of investigation must be explored.

Because of its geometry, measurement methods do not allow visualization of the liquid fragmentation inside the nebulizer. We assumed in this study that numerical simulation could provide results regarding atomization phenomena, which could be valuable for understanding and optimizing the processes. In order to evaluate this assumption and to gain trust into the numerical modeling, it has to be compared to experimental data characterizing the aerosol (diameter and phenomenology): this is the purpose of the present study.

Many studies use numerical models to describe the formation, the motion of droplets and their interaction with solid elements [5], for example in fields like combustion or atmospheric pollution [6], [7]. However, these models cannot be directly applied to drug nebulization, because they assume a pressurized liquid phase flowing into a nozzle, then subject to outside forces (air blast, swirl, ultrasounds). Indeed, liquid fragmentation in a jet nebulizer is caused by air flowing through a nozzle, sucking the liquid inside its container by Venturi effect and then propelling drops fast against an impinging solid surface named baffle [3]. Several physical processes of different scales occur simultaneously, making difficult the prediction of the characteristics of the produced aerosol. Breakup models assume already formed spherical drops. A new and comprehensive model, combining multiphase flow modeling, both dispersed flow as well as free surface flow, is therefore needed to understand and characterize the generation of micrometric droplets through pressurized air, shearing and impingement forces. Multiphase flow modeling and particle tracking are available in commercial CFD software like ANSYS Fluent [8]. In the case of nebulizers, large discrepancies of the air velocity are met, from laminar to supersonic flow, with high fluctuations at the nozzle outlet when both phases interact with each other. Besides, due to the transient behavior of this process as well as the small cell size needed to resolve the turbulent scales and to follow the generation of micrometric droplets, high computation power is mandatory.

After being set-up, the model had to be validated with experimental data. Two criteria were chosen to compare numerical results to experiments: macroscopic phenomena and particle size distribution. Images of air/liquid interfaces obtained numerically inside the domain could be compared to images of liquid behavior, acquired with a fast CCD camera between the nozzle and the baffle. Three different methods were used to obtain particle size of the aerosol cloud produced at the exit of the baffle area. After describing the nebulizer, the numerical model and the experimental methods employed to characterize the aerosol, numerical and experimental results have been compared and analyzed.

## Materials and Methods

### Nebulizer

**Figure 1:**
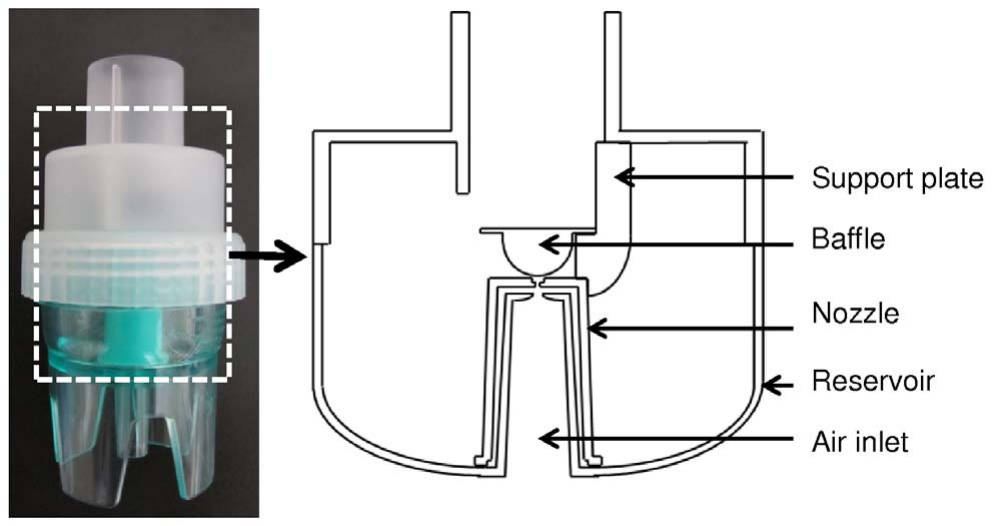
Updraft nebulizer. The different parts of the Updraft nebulizer (Hudson) are displayed, including the nozzle and the baffle modeled in this paper.

A jet nebulizer produces droplets by aspirating a liquid into a nozzle with compressed air through Venturi effect (figure 1). The ejected liquid fragment into droplets then hits a baffle. The Updraft II Optineb (Hudson, Teleflex Medical, NC, USA) nebulizer was chosen to design the geometry of the simulation domain. It has a hemispheric baffle and is axially symmetric. Three support plates are used to ensure the distance between nozzle and baffle. To maintain the symmetry, these plates were not considered in the numerical domain. A 2D-axisymmetric preliminary model was used in order to set physical and numerical models, before using a 3D model. Dimensions of the device were obtained with a digital caliper (accuracy: 20 µm) and internal dimensions of the nozzle were measured via Smartscope (accuracy: 5 µm).

Two air flow rates were considered in that study, 2 L/min and 8 L/min. These values represent the limits of the typical operating range of a nebulizer [9]. The nebulizer was filled with 5 mL water and connected to an air bottle equipped with a regulator to set the flow rate.

### Numerical Study

**Figure 2:**
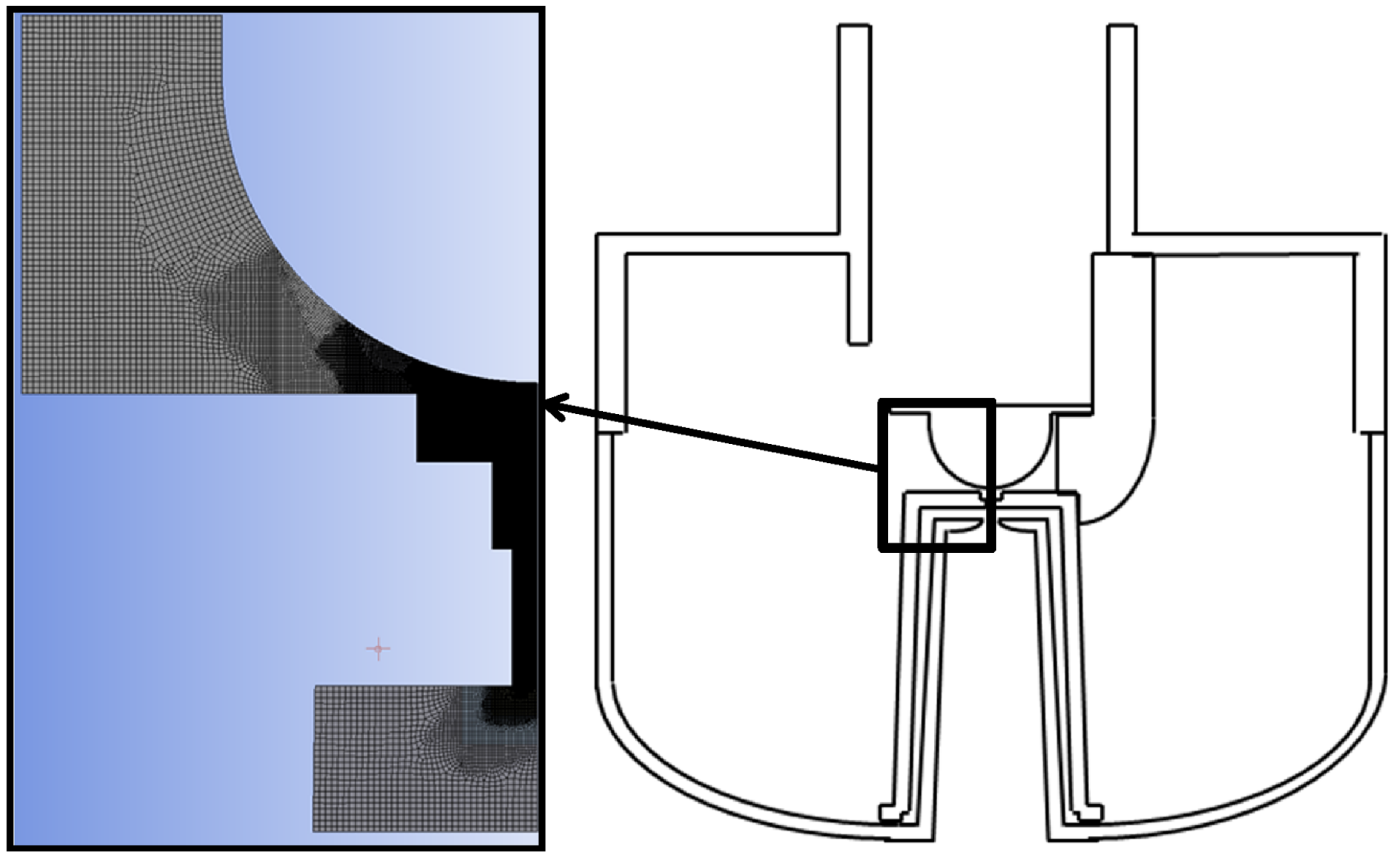
Numerical domain. The numerical domain is based on the geometry of the Updraft nebulizer. The area includes the nozzle exit and the surroundings of the hemispherical baffle.

Both the 2D and the 3D simulation domain were designed with ANSYS Workbench (DesignModeler and Meshing) based on the geometry of the Updraft nebulizer. It includes the areas where the liquid continuous phase turns into a discrete droplet phase, after impacting the hemispherical baffle (figure 2). Because of its axial symmetry, the 3D considered domain could be reduced to a periodic 15° angular sector, decreasing the whole cell amount to a very reasonable 4 million cells. Minimal mesh size was 4 µm in the central zone, where the liquid fragmentation takes place. That size was coarsened to 20 µm in the exit area, where droplets are already created. A combination of structured hexahedral mesh in the fragmentation region and unstructured hexahedral mesh elsewhere has been done to limit the numerical diffusion.

Calculations in a two-dimensional axisymmetric domain were used to determine which physical and numerical models should be used in Fluent 13.0. First a steady-state single-phase case was run with air only. A first set of parameters were selected in Fluent as a basis for the case: discretization numerical schemes, air as a compressible ideal gas and a RANS turbulence model, the Shear Stress Transport (SST) model [10], with low Reynolds option in the 2 L/min case. These calculations allowed the determination of parameters of the airflow inside the domain, mainly pressure and velocity inside the nozzle hole, and confirm the pressure/flow rate relationship, previously evaluated experimentally. When adding a liquid phase a transient calculation was needed, with a very small time step Δt (around 10 ns), due to the high air velocities in the nozzle compared to the mesh size: a Courant number (C = UΔt/Δx, with U the maximum flow velocity and Δx the finest mesh size) close to 1 is indeed required to accurately simulate such physics. The large amount of computational time needed to simulate a few milliseconds of nebulization justifies the use of 2D modeling to set the multiphase case numerically. Relevant modeling parameters in Fluent were selected to predict the behavior of the liquid, the formation and transport of the droplets. A test matrix of settings was used to determine the best trade-off for mesh size, numerical discretization schemes, turbulence modeling and interface tracking modeling. A two-phase flow model was considered, within the Eulerian mixture framework: here the Homogeneous Volume of Fluid model (VOF/free surface) [11], in which liquid and gas phases are considered as continuous media. Both phases share a momentum equation and an energy equation, as well as the turbulence equations. An equation for the transport of volume fraction is written for each phase, the system is then closed through an algebraic relation, the sum of volume fractions being equal to one. A heterogeneous approach where each phase has its own momentum equation has been tested without any major improvement: it was not considered here to lower the computational effort. The gas/liquid interface was obtained via a piecewise linear interpolation explicit scheme (Georeconstruct in Fluent). Each computational cell stores the volume fraction value as well as its gradient to evaluate the slope; the reconstruction of the gas/liquid interface is then a geometrical scheme, hence not a diffusive numerical scheme.

Numerical 2D images could be used to check the predicted phenomena. However, 3D computations are needed to describe with optimal accuracy the generated droplets and the turbulent flow. In 2D, after exiting the nozzle, some of the liquid was accumulated the stagnation point where the spherical baffle crosses the symmetry axis. The liquid then escaped out of the domain through the axis, being probably a numerical artifact, as it was not obtained in a 3D angular domain. This supports then the need of such a 3D model.

In 3D, to get more accurate results, Large Eddy Simulation (LES) with Dynamic Sub-grid model (WALE) was used as the turbulence model to account for the wide range of Reynolds regimes in the process: the use of such advanced Scale-Resolving Simulation (SRS) was not consuming more computation power as the time step is mainly driven by the micrometric droplet transport. At 2 L/min, the Reynolds number (Re = UD/ν, with D the nozzle diameter and ν the kinematic viscosity) is around 4,000 and it is around 16,000 at 8 L/min. With LES, all eddies with a size over the mesh size are resolved and the other ones are modeled inside each cell. This way, for low Re, the turbulent dissipation is not over-estimated as it would be with RANS models. The Pope criterion [12] is traditionally used to assess the relevance of a mesh for LES. It says that the LES is valid if the resolved energy represents at least 80% of total energy in the whole domain. A macro in C language, also called User Defined Function (UDF) was written and run in Fluent to compute the resolved turbulent kinetic energy and the simulated energy on the sub-grid scale. The minimal solved energy ratio, computed inside each cell, was 81% at 2 L/min and 90% at 8 L/min, proving the relevance of the use of LES here.

After setting all relevant parameters, the 3D calculations were run in parallel on supercomputers from the Nuclear Energy Commission (CEA). Fluent was parallelized on 128 cores. The scalability study on the case showed that this repartition was optimal with this mesh size. At 2 L/min the average time step was 60 ns, while at 8 L/min the model was more unstable and the time step was decreased to around 7 ns. Computations were run during five months at 2 L/min and three months at 8 L/min, representing respectively 100 ms and 4 ms. A UDF was developed and compiled to count droplets in the domain and determine their diameter and center position at regular time steps. Values for over 10,000 droplets were compiled for each flow rate on the overall physical time. This number was sufficient to obtain converged values.

### Experimental Methods

Various experimental methods were used to get accurate qualitative and quantitative data to be compared with numerical results. In order to characterize the droplets ejected from the hemispherical baffle of the Updraft, its plastic cover was cut so that laser beams (PDA & Laser diffractometer) and CCD camera could reach the area. The measurements were made as close as possible to the baffle in order to be compared to numerical data.

#### CCD camera

**Figure 3:**
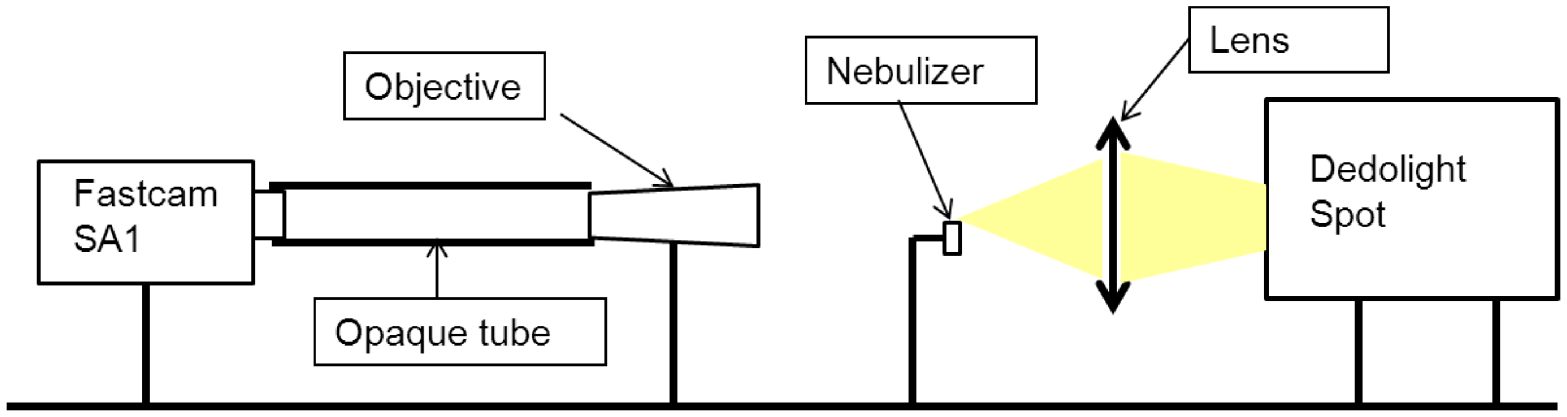
Shadowgraphy with CCD camera setup. A fast CCD camera is aligned with a macro objective, offset at 1

A Fastcam SA1 (Photron) CCD camera was used to acquire high frequency images of liquid fragmentation (films and droplets) into the nebulizer through shadowgraphy. The setup is described in figure 3. All devices are aligned along a graduated axis. A Nikkor 200 mm macro objective was set at 1 m from the camera and a 2x zoom was added at the camera output, providing a magnification of 13.3. Recorded images have a resolution of 1.35 µm/px. The shutter time had to be set at a minimum, 1 µs, because of the relatively high velocity of liquid droplets. This imposed a strong lighting device to compensate the shutter time. A 400 W Dedolight HMI spot followed by a convergent lens was set in the alignment. It provided high brightness in a concentrated area. The nebulizer position was set with micrometric tables in three dimensions so that the acquisition was taken at the exit of the spherical baffle. The acquired images were recorded through Fastcam Viewer. After many tests to determine the best compromise between field size and frame rate, this parameter was set at 10,000 images/s for the 2 L/min case (Resolution: 768×768), and 30,000 images/s (512×352) for 8 L/min, to account for the higher droplet velocity. Image contrast and brightness were adjusted. This software uses its shadow processing module to analyze the grey level pattern and determine the contour of liquid masses and then obtain their size. The grey level gradients were magnified by subtracting from the image the mean value of the set, thus reducing the background noise. The grey level and gradient thresholds above which the droplets had to be taken into account was determined, so that considered droplets had clean boundaries on the screen. This method considers the actual shape of the droplet and provides an equivalent diameter (diameter of the sphere with the same surface area on the image). Overall 5,000 droplets were considered at 2 L/min and 10,000 at 8 L/min. The distributions matched between various sets of images, so these amounts were sufficient to assume convergence. With the available resolution, droplets over 5.2 µm could be recorded. Then size distribution histograms could be plotted. The shadowgraphy method has the advantage of using a similar post-processing method as the numerical analysis, by determining the contour of droplets according to respectively grey level and volume fraction values [13]. Shadowgraphy also allowed the visualization and recording of the macroscopic phenomenology of the liquid in the transient phase.

#### Laser diffractometry

**Figure 4:**
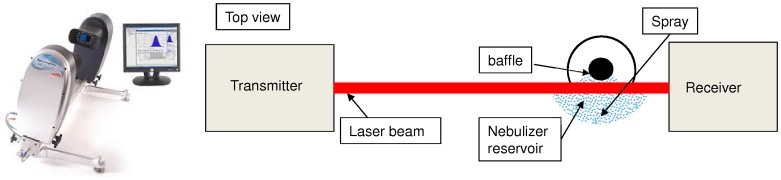
Laser diffractometry setup (Spraytec). On the left is a picture of the Spraytec laser diffractometer. On the right is the experimental setup used. The nebulizer is placed at the crossing of the laser beam, close to the receiver lens. The diffractometer then analyses the spray exiting from the nebulizer baffle.

Laser diffraction is mostly used in aerosol therapy to characterize the particle size distribution produced by a nebulizer [14]. It is well suited for the usual size range of medical aerosol devices, from micrometric to millimetric particles. Diffractometers compute the volume distribution of a spray using the Mie theory, which links a particle size to a diffraction angle. The analysis of the diffraction pattern through a multi-scattering algorithm provides a distribution of the geometric diameter of droplets. In this study, particle size was measured by the Spraytec (Malvern). Following parameters were set in the software: the dispersion code was polydisperse, multi-scattering algorithm was activated and refraction index set for water. The nebulizer was placed on a support so that the 1 cm laser beam crossed the exit area at the extremity of the baffle (Figure 4). The axial symmetry of the produced distribution was supposed. The device had to be placed at 10 cm from the receiver to avoid the contamination of the lens. For each flow rate, 6 acquisitions were made and then the distributions were averaged. Because of the volume distribution, the obtained results could not be compared with the other methods, so the distributions had to be first converted into number distributions. However this conversion magnifies potential errors and should only be used for method comparisons. The measurement range of the Spraytec allows the detection of droplets down to 0.5 µm. The Mie theory assumes all measured droplets to be spherical, which can lead to sizing errors. The diameter obtained through laser diffraction matches the geometric diameter, which corresponds to the diameter of the sphere including the droplet.

#### Phase Doppler Anemometry

**Figure 5:**
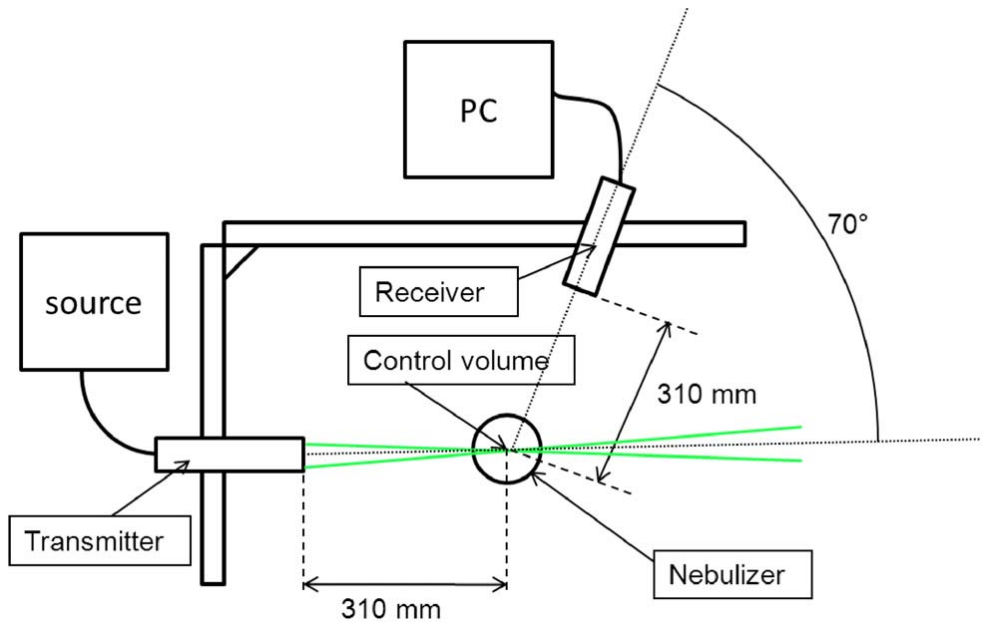
PDA setup (Dantec). The setup described corresponds to the Dantec Dynamics PDA system. The nebulizer is placed at the crossing of the two emitted laser beams. The receiver is placed at 70° off-axis to analyze the interference fringes and extract the size distribution.

Phase Doppler Anemometry is often used to measure simultaneously size of droplets passing in a definite point [15]. This method is used in various domains like diesel sprays and also sometimes in medical sprays [16]. The PDA method measures the phase shift between two laser beams crossing into a small control volume where droplets flow. The diameter of the droplet is directly related to the phase shift and its velocity depends on the frequency shift. This is a localized measurement; each particle crossing the control volume is counted and analyzed. The PDA system produced by Dantec Dynamics was used in this study. Each acquisition considers 10 000 droplets. Like the other experimental methods, the nebulizer was set on a support so that the baffle exit matches the crossing of the beams (Figure 5). It was connected to a compressor equipped with a regulator, providing the appropriate flow rates. Acquisitions were made at different positions within the exit area in order to verify the spatial homogeneity of the produced aerosol. This method provided directly a number distribution. Several masks can be used with the PDA system, restricting the measurement size range. The mask for large particles (up to 300 µm showed no particles, so the smallest mask was used, allowing a detection of droplets from about 1 to 80 µm. With PDA, only spherical droplets are considered in the provided distribution.

## Results

### Phenomenology

**Figure 6:**
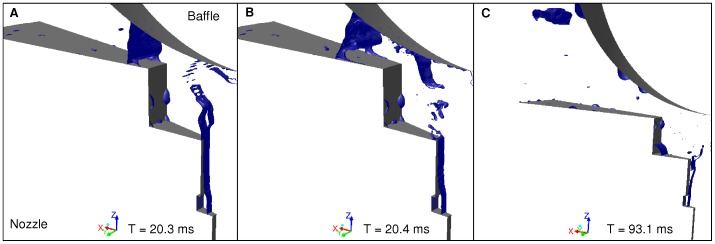
Free surface images at 2/min. Film formation (A), Film Stretching (B), Droplet formation (C). The images display the numerical results in terms of liquid phenomenology at 2 L/min. The free surface, corresponding to the interface between air and water, is displayed in blue. On image A, the formation of a liquid film can be observed. The film stretches until it breaks up (image B). A large droplet is then formed (image C). A new cycle then follows.

**Figure 7:**
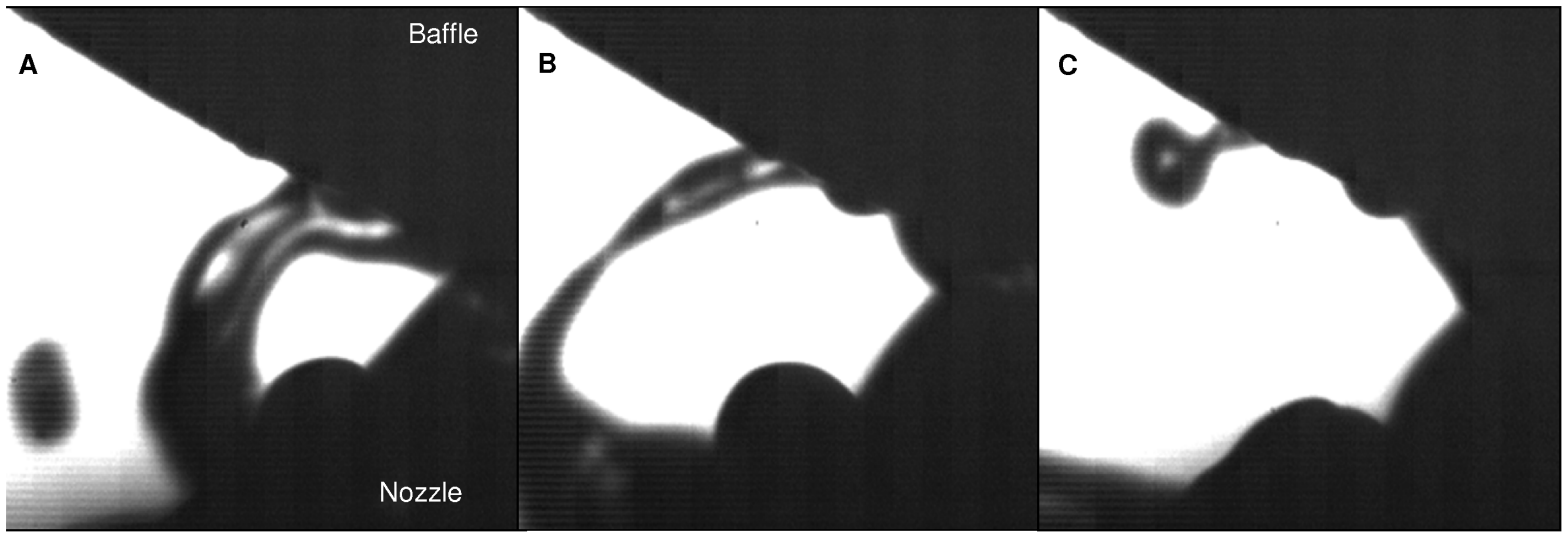
CCD camera images at 2/min: Film formation (A), Film stretching (B), Droplet formation (C). The images display the results obtained with the shadowgraphy method from the CCD camera between the nozzle and the baffle at 2/min. On image A, the formation of a liquid film can be observed. The film stretches until it breaks up (image B). A large droplet is then formed (image C). A new cycle then follows.

**Figure 8:**
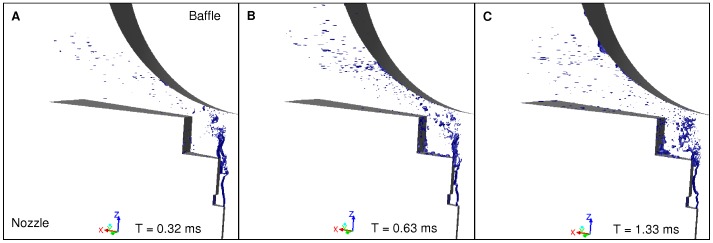
Free surface images at 8/min: Expulsion of droplets (A), Spray densification (B), Annular liquid layer (C). The images display the numerical results in terms of liquid phenomenology at 8/min. The free surface, corresponding to the interface between air and water, is displayed in blue. A continuous flow of droplets is expelled from the area between nozzle and baffle (A). The density of the spray is variable (image B). Then the droplets on the baffle begin to agglutinate and form an annular liquid layer (image C).

**Figure 9:**
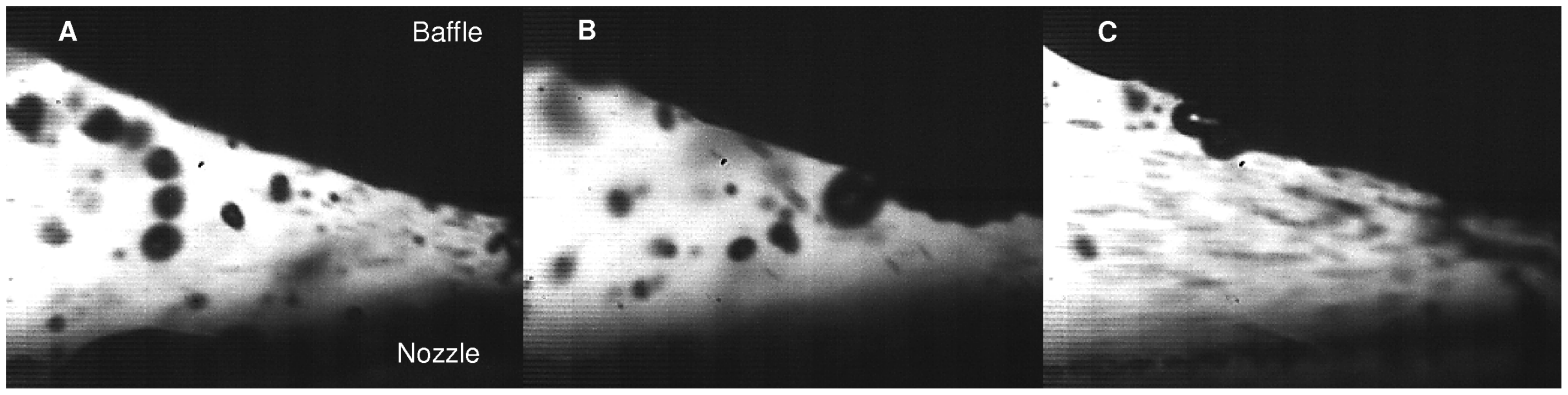
CCD camera images at 8/min: Expulsion of droplets (A), Spray densification (B), Annular liquid layer (C). The images display the results obtained with the shadowgraphy method from the CCD camera between the nozzle and the baffle at 8/min. A continuous flow of droplets is expelled from the area between nozzle and baffle (A). The density of the spray is variable (image B). Then the droplets on the baffle begin to agglutinate and form an annular liquid layer (image C).

Regarding liquid film break-up phenomenology during the nebulization process, both numerical results and camera observations showed similar patterns. Figures 6 and 7 show respectively numerical and experimental results at 2 L/min, whereas figures 8 and 9 show results at 8 L/min. Numerical images (Figures 6 and 8) display the gas/liquid interface between the nozzle and the spherical baffle, which corresponds to cells with a volume fraction of 0.5, representing the contour of liquid films and droplets. Experimental images (Figures 7 and 9) were obtained with the CCD camera.

At 2 L/min, the air flow had low compressibility (The Mach number, defined as the ratio of air velocity over sound velocity, is Ma = 0.3) and low turbulence (Re = 4,000). Surface tension effects could be observed to maintain the liquid into large liquid masses. The occasional formation of liquid films between the baffle and the nozzle was observed in numerical and experimental results (Figures 6 and 7). Many droplets impinged the baffle, then spread and coalesced to form larger liquid masses on the solid surface. They were then subjected to a force balance between surface tension and air inertial forces. When the mass became thick enough, it slid towards the central axis due to gravity and formed a film bridging nozzle and baffle. Then the airflow blew up the film into many droplets with size up to 150 µm. According to simulation results, this process was periodic and the majority of droplets were produced during the film breakup. Each occurrence of the formation of a liquid film was separated by around 23 ms. Camera images showed no noticeable periodicity. However, it seemed that each phenomenon could be observed at different times and angular positions. According to numerical results, a small amount of droplets could be generated through rebound or breakup of liquid masses with high velocity on the lower part of the baffle surface, perpendicular to the airflow. Numerical images allow an exclusive access to the fragmentation process, which could not be correlated with experimental methods due to its location inside the nozzle. Obtained images showed a periodic wave on the interface between liquid and air, where the liquid exits the nozzle and encounters the 100 m/s air flow. Waves had approximately a periodicity of 0.9 ms. Due to air motion, waves hit the nozzle internal wall, causing the generation of droplets with various sizes (sometimes several hundred microns) and shape factors (from spherical droplets to liquid strings).

Numerical and experimental results at 8 L/min are respectively displayed on figures 8 and 9. At this flow rate, air velocity in the nozzle can get up to 600 m/s and is mainly supersonic (Ma ∼ 1.4). Turbulence was higher (Re = 16,000). The liquid masses coming from the nozzle hit the baffle with higher velocity and smaller size, most of which did not reach the sphere. Droplets were continuously expelled from the nozzle hole (figures 8a and 9a). A steady thin annular layer of liquid was progressively formed on the sphere (figures 8c and 9c). Unlike the low flow rate case, no liquid film was observed between nozzle and baffle surfaces. In that case, an axial symmetry of the phenomena was observed. The volume of liquid contained within the meshed domain was stable after around 2 ms, showing that the transient phase was insignificant at 8 L/min. 4 ms were computed, which was sufficient to get an overall view of phenomena occurring in this case.

Regarding atomization, Kelvin-Helmholtz waves were obtained at the air-liquid interface in the nozzle hole but they occurred at much faster rate (about every 0.1 ms). A large amount of droplets were produced during that phase, before baffle impingement. Unlike the low flow rate case, the liquid was not blocked in the step cavity due to the airflow because of its higher velocity.

### Particle Size

In order to make a relevant comparison, all size distributions are number distributions considering only droplets over 5 µm. So, droplets under 5 µm were excluded from PDA and laser diffraction distributions.

**Figure 10:**
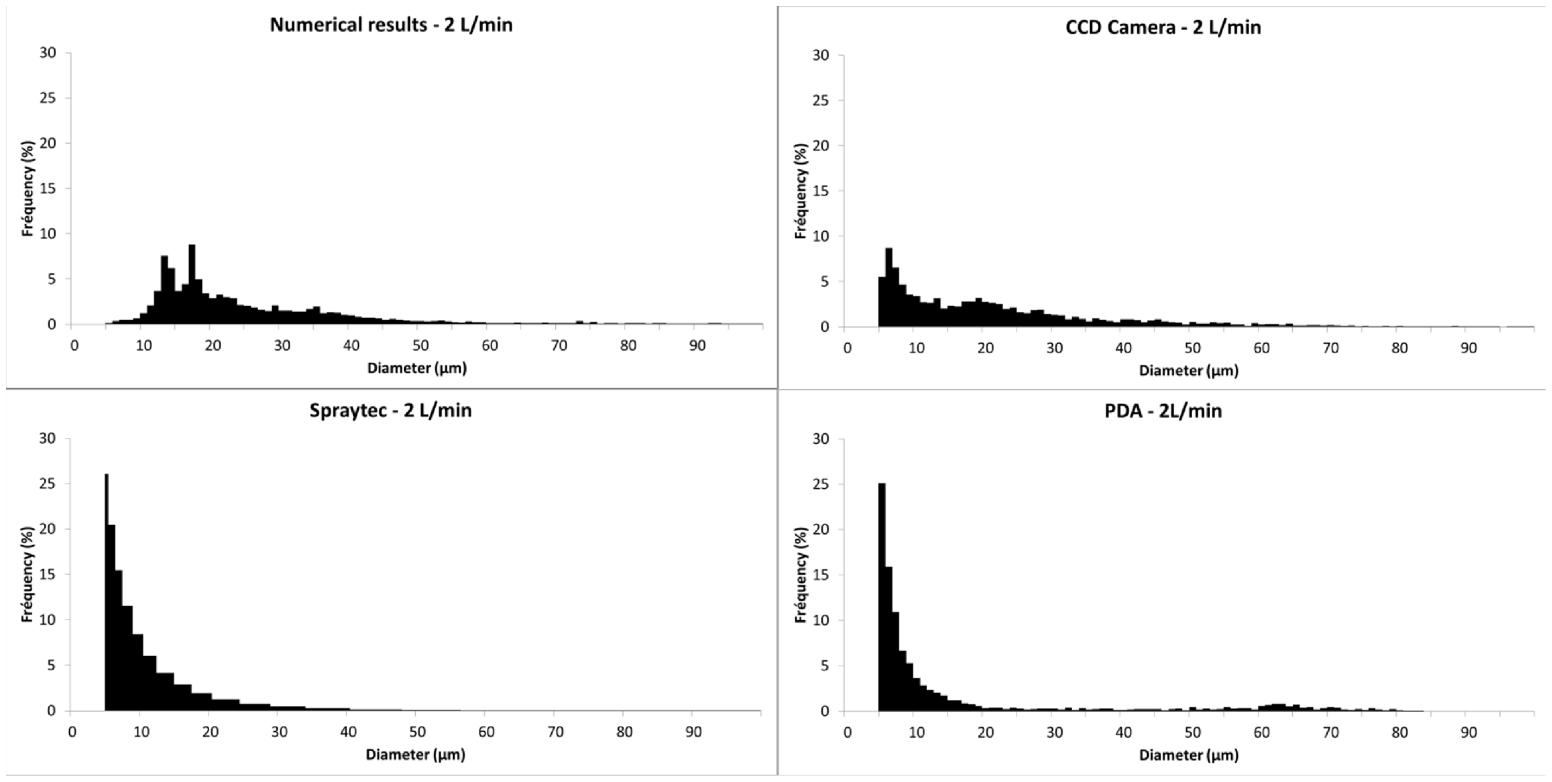
Particle size distributions at 2/min. The size distributions obtained with the numerical results and the experimental methods at 2/min are represented. These are number distributions with a size range going from 5 to 100 µm. Numerical and Shadowgraphy results are similar whereas Laser Diffraction and PDA provide much smaller diameter values.

**Figure 11:**
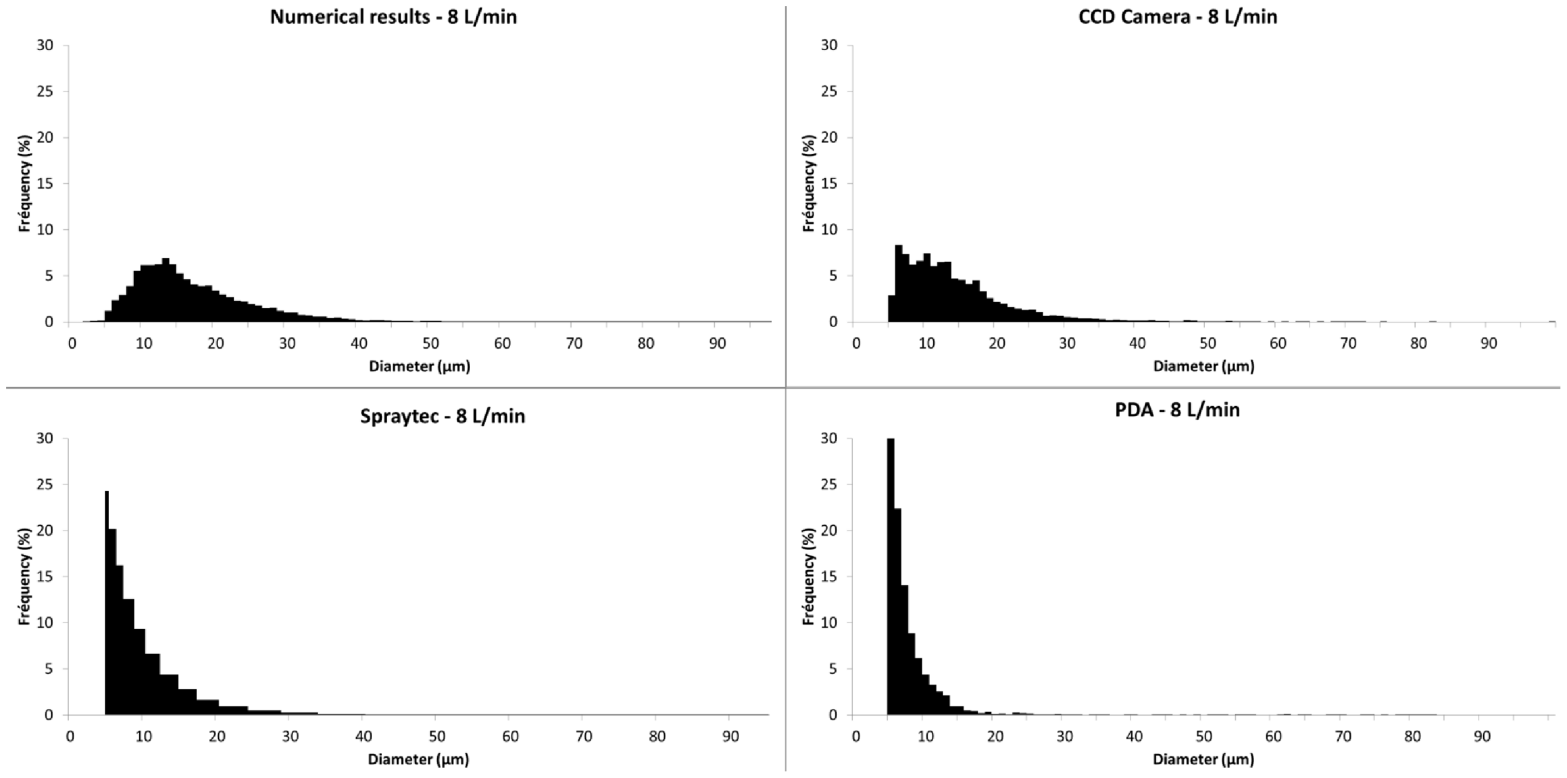
Particle size distributions at 8/min. The size distributions obtained with the numerical results and the experimental methods at 8/min are represented. These are number distributions with a size range going from 5 to 100 µm. Numerical and Shadowgraphy results are similar whereas Laser Diffraction and PDA provide much smaller diameter values.

Table 1 shows the median diameter D_50_ and span factor obtained from numerical results and experimental droplet size measurement methods, for 2 L/min and 8 L/min respectively. The span factor is defined as (D_90_–D_10_)/D_50_, with D_x_ representing the diameter under which x % of the droplets are. For each given value, more than 10,000 droplets were considered to ensure convergence. For all methods, the median droplet size was lower and the distribution less spread at a higher flow rate, except laser diffraction where low and high flow rate sizes are similar. Numerical simulation and experimental shadowgraphy consider the same size range and have similar limitations (mesh size/image resolution): they also provided similar values. The difference between the median values was about 20% and the spreading of the distributions was similar. PDA and laser diffraction median sizes were significantly different from the numerical value. Figures 10 and 11 show particle size distributions obtained with the simulation and the three measurement methods. On shown histograms for both these methods, particles with a diameter smaller than 5 µm were removed from the results and further calculations, so that distributions could be compared in a similar size range. For both flow rates, the numerical and camera plots can be considered as similar. Distributions followed the same pattern. PDA and laser diffractometry provided much smaller values. Their distributions didn’t then match numerical results. However the amount of droplets over 5 µm represented only a small fraction of measured droplets in terms of number (30% for PDA and around 5% for laser diffraction) and the amount of droplets over 20 µm was insignificant, in opposition to sizes obtained numerically.

**Table 1:** Particle size obtained with numerical simulation and experiments - Median diameter (Span).

	Numericalresults	Shadowgraphy	PDA	Laserdiffraction
2 L/min	19.7 µm(1.7)	17 µm(2.4)	6.9 µm(7)	7.1 µm(1.4)
8 L/min	14.4 µm(1.4)	11.8(1.5)	5.9 µm(1.1)	7.3 µm(1.2)

## Discussion

The numerical model could predict the liquid film behavior phenomena observed experimentally. Indeed, for both tested air flow rates, which characterize two different turbulence regimes, images obtained from the numerical model could be well matched with the ones obtained with the fast CCD camera. At 2 L/min, surface tension forces tended to compensate inertial forces due to the air flow. The Weber number, defined as the ratio of inertial forces applied on a droplet over surface tension forces (We = ρU^2^d/γ, with ρ the air density, d the diameter of the droplet and γ the surface tension), was around 10 for large droplets exiting the nozzle hole with a diameter around 100 µm, meaning that surface tension effects of the liquid could not be neglected. It could explain that these droplets keep their integrity and could spread on the spherical baffle. They were finally dragged along the surface through airflow inertia. At this point, a liquid film could form with the nozzle upper surface. At 8 L/min, the force balance was different. Air inertial forces are dominant over surface tension. Weber number was over 100 for droplets ripped off from the primary liquid layer. When droplets are spread, they are exposed to high velocity gradients and could be ripped-off through shear flows. However, a relatively steady annular liquid mass could still be formed on the sphere with remaining liquid masses spread on the surface. Atomization inside the nozzle followed also a Kelvin-Helmholtz pattern due to a high velocity gradient on the air/liquid interface.

Despite its good prediction on qualitative criteria, the numerical model has also to be validated with quantitative data on particle size distribution. Laser diffraction, PDA and Shadowgraphy use different principles and procedures to get a size distribution, with various size ranges and processing methods. Regarding droplets over 5 µm, distributions obtained with laser diffraction and PDA didn’t match numerical distributions at both low and high flow rate. The difference is lower at high flow rate because of a less dispersed distribution.

Regarding laser diffraction, the diffraction angle is directly dependent upon the geometric diameter of the droplet. This value may be wrong in case of non-spherical droplets. This method usually characterizes nebulizer performance at the exit of the complete nebulizer, where large droplets are previously selected and recycled. Droplets have reached their final size and spherical shape. Furthermore, the necessity for the volume distribution provided by the Spraytec to be converted into a number distribution for comparison with other distributions could also cause divergences. For larger particles, high values in volume corresponds to a very little amount of droplets, thus implying negligible drops number in the larger size range.

PDA analyzes droplet size by measuring the phase shift between two laser beams. This shift depends on the variation of optical path caused by the droplet for the beam to reach the receiving lens. This path depending on the curvature radius, the sphericity of the droplet is necessary to get a reliable value. Our PDA system checks the sphericity and then excludes non-consistent droplets from the distribution, 30% in our case. Nevertheless, smaller droplets reach a spherical shape faster than larger ones due to lower influence of surface tension. Therefore, the PDA system tends to exclude a higher amount of large drops.

All of these limitations could provide explanations for the noticed bias. Therefore, laser diffraction and PDA could not be employed to validate our numerical model. Unlike laser diffraction and PDA, the numerical method and the shadowgraph method have to post-process data to get a size distribution histogram. Both methods provide images at a defined time-step, where the presence of liquid in the domain or field of view is displayed by a grey level scale in a pixel (CCD camera) or a volume fraction value in a mesh cell (Numerical model). Then, the gradient of this value is used to get air/liquid interface and draw droplet contours. At both flow rates, the distribution plots are similar. The model overestimates the median size only by 20%. The numerical size determination UDF is based on droplet contours, which correspond to the iso-value curve for a volume fraction of 0.5. The uncertainty on boundaries highly depends on the cell size, 4 µm. Furthermore the UDF counts and measures every liquid mass within the model, including droplets spread on the solid surfaces. In the same way, to evaluate the droplet size from CCD camera images, Dynamicstudio use a grey level gradient and a threshold set by the user to determine the presence or not of liquid in a pixel. In our camera setup, contours are blurry due to a very narrow depth of field. The precision of diameters obtained through this method corresponds then to about 1 or 2 pixels (2–3 µm). The resolution differences could explain the slight overestimation of the size distribution by the model. The shadowgraph method seems to be the most appropriate method to validate our numerical model. However, the validation can be considered as reliable only for droplets whose size is accurately estimated by the model, which means with a diameter corresponding to at least 2–3 cells. Therefore, our model gives accurate size estimation for droplets with a diameter down to than 10 µm.

Our model aimed at eventually optimizing nebulizer devices, whose requirement is to maximize the flow rate of droplets with a diameter smaller than 5 µm. In order to characterize these sizes with the current Eulerian free surface modeling strategy that was adopted here, we would need to divide at least by two the minimum cell size, which would theoretically increase computing time eightfold.

Droplet size distributions provided by laser diffraction on the overall size range (1 µm to around 150 µm) showed continuous log-normal distributions. The physical laws and phenomena causing liquid masses and layers to breakup into smaller droplets can then be supposed continuous between droplets from 1 to 10 µm and larger ones. So, if a parameter change in the model implied an optimization of the output in the larger size range, the overall actual output would also be optimized.

Furthermore, even if the fragmentation of liquid into droplets within that order of magnitude could not be accurately visualized and characterized numerically, the model offered valuable information from which their phenomenology could be deduced. The model offers the ability to visualize phenomena inside the nozzle, where no experimental method could access. This gives directions to optimize the internal design of the nebulizer.

## Conclusion

In conclusion, a 3D numerical model for predicting behavior and output characteristics of a medical nebulizer device has been set with ANSYS Fluent from a defined geometry. Results in terms of macroscopic phenomena and particle size have been validated with data obtained through shadowgraph method with a fast CCD camera. Usual spray characterization methods like laser diffraction and PDA were not adapted for comparison with the model due to the method hypotheses and size ranges. A better precision of the model, meaning an even more accurate prediction of smaller sizes could be achieved by reducing the mesh size with higher computation time. Another strategy would be to couple the Eulerian free surface model with the Lagrangian model (Discrete Phase Model in Fluent) from a pre-defined droplet size threshold to lift the mesh cell size limitation.

Although considerable computation times prevent a direct numerical optimization, this model helps the understanding of nebulization phenomena and provides information for design suggestions of the nozzle/baffle parts. In order to optimize a whole nebulizer device, the design must then be completed by droplet transport considerations through the selection process.
